# Associations between Vascular Endothelial Growth Factor Gene Polymorphisms and Different Types of Diabetic Retinopathy Susceptibility: A Systematic Review and Meta-Analysis

**DOI:** 10.1155/2021/7059139

**Published:** 2021-01-04

**Authors:** Liming Hu, Chunmei Gong, Xiaoping Chen, Honghao Zhou, Junxia Yan, Wenxu Hong

**Affiliations:** ^1^Shenzhen Center for Chronic Disease Control, 2021 Buxin Road, Luohu District Shenzhen 518020, China; ^2^Department of Epidemiology and Health Statistics, XiangYa School of Public Health, Central South University, Changsha, China; ^3^Hunan Provincial Key Laboratory of Clinical Epidemiology, XiangYa School of Public Health, Central South University, Changsha, China; ^4^Institute of Clinical Pharmacology, Central South University, Changsha, China

## Abstract

**Background:**

Vascular endothelial growth factor (*VEGF*) gene polymorphisms have been shown to be associated with the risk of diabetic retinopathy (DR), but the results were inconsistent. The aim of this study was to systematically assess the associations between *VEGF* gene polymorphisms and different types of DR (nonproliferative DR and proliferative DR).

**Methods:**

Electronic databases PubMed, Embase, Web of Science, CNKI, and WANFANG DATA were searched for articles on the associations between *VEGF* gene polymorphisms and different types of DR up to November 6, 2019. Pooled odds ratios (ORs) and 95% confidence intervals (CIs) were calculated, and subgroup analyses were conducted by ethnicity. Sensitivity analysis was conducted to assess the stability of the results. Publication bias was assessed by using the Egger regression asymmetry test and visualization of funnel plots. A systematic review was conducted for polymorphisms with a high degree of heterogeneity (*I*^2^ > 75%) or studied in only one study.

**Results:**

A total of 13 and 18 studies analyzed the associations between *VEGF* SNPs and nonproliferative DR (NPDR) as well as proliferative DR (PDR), respectively. There were significant associations between rs2010963 and NPDR in Asian (dominant model: OR = 1.29, 95%CI = 1.04 − 1.60); and rs2010963 is associated with PDR in total population (dominant model: OR = 1.20, 95%CI = 1.03 − 1.41), either Asian (recessive model: OR = 1.57, 95%CI = 1.04 − 2.35) or Caucasian (recessive model: OR = 1.83, 95%CI = 1.28 − 2.63). Rs833061 is associated with PDR in Asian (recessive model: OR = 1.58, 95%CI = 1.11 − 2.26). Rs699947 is associated with NPDR in the total population (dominant model: OR = 2.04, 95%CI = 1.30 − 3.21) and associated with PDR in Asian (dominant model: OR = 1.72, 95%CI = 1.05 − 2.84).

**Conclusions:**

Rs2010963, rs833061, and rs699947 are associated with NPDR or PDR, which may be involved in the occurrence and development of DR.

## 1. Introduction

With the change of modern people's lifestyle, diabetes mellitus (DM) has become a serious public health problem in the world, which has seriously affected the life quality of patients and brings huge medical and economic burden to the society [1, 2]. Diabetic retinopathy (DR), one of the most common microvascular complications of DM, is the leading cause of acquired blindness in middle-aged people [3]. The pathophysiological mechanism of DR is complex, and there are no effective treatments at present. According to the progress and severity of DR, it can be divided into nonproliferative DR (NPDR) and proliferative DR (PDR), whose pathogenesis and pathophysiology were not exactly the same [4]. Until now, the pathogenesis of this disease has not been fully understood; it was generally believed that the occurrence of DR is affected by many factors, including age, inflammation, hypoxia, and genetic factors [5, 6]. In recent years, with the development of genome-wide association study (GWAS), the risk loci associated with DR discovered by GWAS study have provoked researchers' attention and research on the genetic factors of DR, provided a theoretical basis for understanding the role of genetic factors in the occurrence, prevention, and treatment of DR.

Vascular endothelial growth factor (VEGF) is a special type of cytokine, which is secreted by various cells such as vascular endothelial cells and smooth muscle cells, and plays an important role in regulating blood vessel formation, tumor growth and development and atherosclerosis [7]. Study has shown that VEGF is a basic regulator of normal and abnormal angiogenesis, and it is the key mediator of many angiogenesis-related diseases [8]. Intraocular neovascularization mediated by VEGF may lead to vitreous hemorrhage, retinal detachment, and eventually blindness [9], which is closely related to the occurrence and development of DR. Evidence showed that serum VEGF levels significantly elevated in patients with DR [10]; some factors that affect the occurrence of DR may be achieved by regulating the expression of *VEGF* [11, 12], suggesting that VEGF may play an important role in the development of DR. The human *VEGF* gene is located on chromosome 6p21.3, whose single nucleotide polymorphisms (SNPs) can affect gene expression by altering key regulatory sequences or by altering mRNA stability at key regulatory loci [4]. In recent years, a large number of studies have investigated the association between *VEGF* gene polymorphisms and NPDR or PDR, but the results were inconsistent [13–15]. The contradictory results may be due to ethnic differences, small sample size, clinical, or method heterogeneity.

Meta-analysis can increase the statistical efficiency by quantitatively synthesizing the results of individual studies [16]. Although some studies have done meta-analysis between *VEGF* gene polymorphisms and DR [4, 17–19], there were differences among the results, and they only analyzed the association between *VEGF* SNPs and DR in general without distinguishing NPDR or PDR. In addition, some new studies on the association of *VEGF* SNPs and different types of DR have been published recently. Therefore, in this study, we completed an updated systematic review and meta-analysis to evaluate the association between *VEGF* SNPs and NPDR as well as PDR.

## 2. Materials and Methods

This meta-analysis was performed according to the recommendations for improving the quality of meta-analyses of genetic association studies and in accordance with the guidelines of the Human Genome Epidemiology Network [20]. Evaluation of studies' quality was based on the Strengthening the Reporting of Genetic Association Studies guidelines [21]. This systematic review and meta-analysis was conducted in accordance with the PRISMA reporting specification (Supplementary 1).

### 2.1. Literature Search Strategy

We systematically searched the electronic databases (PubMed, Embase, Web of Science, CNKI, and WANFANG DATA) to identify articles on the association between *VEGF* SNPs and different types of DR that have been published up to November 6, 2019. The terms used for search were (“diabetic retinopathy∗” OR “diabetes retinopathy∗” OR “DR∗”) AND (“vascular endothelial growth factor∗” OR “vascular endothelial cell growth factor∗” OR “VEGF∗”) AND (“gene∗” OR “polymorphism∗” OR “mutation∗” OR “single nucleotide polymorphism∗” OR “SNP∗” OR “variant∗”) (Supplementary 2). The reference lists of relevant articles were checked to identify additional eligible studies not included.

### 2.2. Inclusion and Exclusion Criteria

Eligible articles included studies assessing the association of *VEGF* gene polymorphisms with confirmed NPDR or PDR in humans. The inclusion criteria were as follows: (1) case-control studies investigating the association between *VEGF* SNPs and NPDR or PDR; (2) enough genotype data were available to calculate odds ratios (ORs) and 95% confidence intervals (CIs); and (3) both type 1 DM and type 2 DM met the inclusion criteria. The exclusion criteria were as follows: insufficient information for available data, reviews, comments, meeting abstracts, animal models, and case report. If duplication or overlapping data occurred, only the study that included the largest individuals was included.

### 2.3. Data Extraction and Quality Assessment

Two authors (LM Hu and CM Gong) independently extracted the data from the included studies, and disagreements were resolved by consensus with a third author (JX Yan) and discussion. The following characteristics were extracted from the selected studies: the first author, year of publication, country, ethnicity, sample size, mean age, sex, duration of diabetes, numbers or frequencies of genotypes and alleles, and the Hardy–Weinberg equilibrium (HWE) status (obtained from the article or calculated by genotype distributions). Ethnicity was classified as Asian and Caucasian. The Newcastle–Ottawa quality assessment scale (NOS) was used to assess the quality of studies included in this meta-analysis. Studies with NOS scores ≥ 6 were regarded as high quality.

### 2.4. Statistical Analysis

The distribution of genotypes and alleles between case and control group was compared by the Chi-square test. Pooled ORs and corresponding 95% CIs were calculated to evaluate the strength of association by using a fixed-effect model or random-effect model. The significance of pooled OR was determined by the *Z* test. The pooled results were evaluated by the dominant model, recessive model, and allelic model. The heterogeneity between studies was assessed using *I*^2^ statistic and corresponding *P* value. When *P* < 0.1 or *I*^2^ > 50%, significant heterogeneity was considered, and the random-effect model was used. Otherwise, the fixed-effect model was used. Subgroup analyses were performed by ethnicity. In addition, if there was a high degree of heterogeneity (*I*^2^ > 75%) [22] or identified only in one study that quantitative synthesis was not suitable, the data will be summarized and presented in a descriptive way. Sensitivity analysis was performed for the SNPs with more studies (≥7) to estimate the stability of the results. Potential publication bias was assessed by funnel plots of Begg's rank correlation method and Egger's regression asymmetry. All statistical analyses were performed using the STATA 12.0 software (Stata Corporation, College Station, TX, USA). *P* < 0.05 was considered statistically significant.

## 3. Results

### 3.1. Characteristics of Included Studies

**Figure 1:**
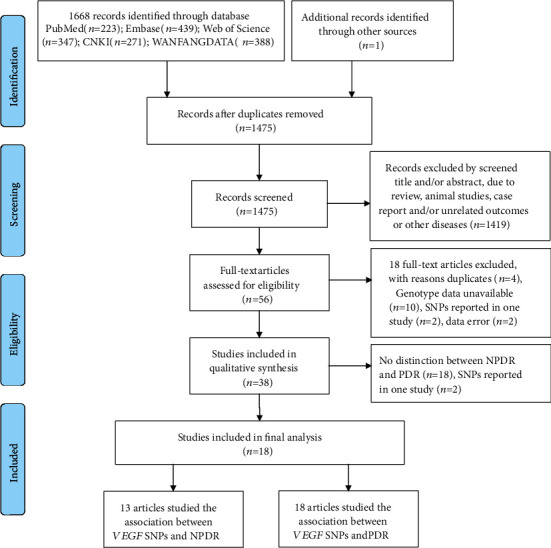
PRISMA flow diagram of study selection process.

A total of 1668 studies were retrieved in the initial search, and 1 study was identified through the relevant references check. After the duplication was removed, 6 SNPs of 18 studies were finally included. The flow chart describing study selection process is shown in Figure 1. There were 13 studies including the association between *VEGF* SNPs and NPDR [13–15, 23–32], and 18 studies including the association between *VEGF* SNPs and PDR [13–15, 23–37]. The characteristics of included studies are summarized in Tables 1 and 2, respectively.

### 3.2. Association between *VEGF* SNPs and Different Types of DR

#### 3.2.1. *VEGF* rs2010963 and the Risk of Different Types of DR

Nine studies including 959 cases and 1357 controls investigated the association between rs2010963 and NPDR, in which 7 studies were conducted in Asian and 2 in Caucasian. Twelve studies including 1431 cases and 2126 controls investigated the association between rs2010963 and PDR, in which 8 studies were conducted in Asian and 4 in Caucasian.

There was statistically significant association between rs2010963 polymorphism and NPDR in Asian population (dominant model: OR = 1.29, 95%CI = 1.04 − 1.60, *P* = 0.023; recessive model: OR = 1.48, 95%CI = 1.11 − 1.99, *P* = 0.009; allelic model: OR = 1.26, 95%CI = 1.08 − 1.47, *P* = 0.003) (Table 3). The CC genotype and C allele of rs2010963 were positively associated with PDR in total population (dominant model: OR = 1.20, 95%CI = 1.03 − 1.41, *P* = 0.022; recessive model: OR = 1.66, 95%CI = 1.21 − 2.28, *P* = 0.002; allelic model: OR = 1.23, 95%CI = 1.11 − 1.37, *P* < 0.001), either Asian (recessive model: OR = 1.57, 95%CI = 1.04 − 2.35, *P* = 0.030; allelic model: OR = 1.26, 95%CI = 1.01 − 1.57, *P* = 0.039) or Caucasian (recessive model: OR = 1.83, 95%CI = 1.28 − 2.63, *P* = 0.001; allelic model: OR = 1.31, 95%CI = 1.09 − 1.57, *P* = 0.004) (Table 4).

Two studies of rs2010963 polymorphism and NPDR in the Caucasian population were conducted in Egypt and Poland, respectively, and their genotype distribution in the case group was opposite, leading to greater heterogeneity between studies. There was no association between rs2010963 and NPDR in Egypt. However, the CC genotype and C allele frequency in NPDR group was significantly higher than that in the control group in Poland (Table 1).

#### 3.2.2. *VEGF* rs3025039 and the Risk of Different Types of DR

Five studies investigated the association between rs3025039 and NPDR as well as PDR. There was a high degree of heterogeneity between studies, so we made a descriptive summary of the results. Studies showed that the T allele of rs3025039 was negatively associated with NPDR and PDR in Chinese population, but it was positively associated with NPDR and PDR in South Korea. Rs3025039 was associated with PDR in Japan. No association was found between rs3025039 and NPDR in India. However, the association between rs3025039 and PDR in India lacks a unified conclusion (Tables 1 and 2).

#### 3.2.3. *VEGF* rs833061 and the Risk of Different Types of DR

Four studies investigated the association between rs833061 and NPDR risk. They were conducted in Pakistani, Chinese, and Polish. The results showed that rs833061-C was positively associated with NPDR in Pakistani, but the direction of the results was inconsistent among Chinese. No association was found in Polish (Table 1).

Five studies with 759 cases and 1093 controls that investigated the association between rs833061 and PDR risk were included. Significant association was found between rs833061 polymorphism and PDR in recessive model in Asian population (OR = 1.58, 95%CI = 1.11 − 2.26, *P* = 0.012) (Table 4).

#### 3.2.4. *VEGF* rs1570360 and the Risk of Different Types of DR

A total of four studies with 559 cases and 927 controls involving rs1570360 polymorphism and NPDR risk were included in this meta-analysis. No association was found between rs1570360 and NPDR (Table 3).

Five studies studied the association between rs1570360 polymorphism and PDR; there was a high degree of heterogeneity that was not suitable for merging the results. These studies found that the A allele of rs1570360 was associated with PDR risk in the Indian and European population, while it had a negative association in the Pakistani population. However, no association was found between rs1570360 and PDR in Korean and Japanese (Table 2).

#### 3.2.5. *VEGF* rs699947 and the Risk of Different Types of DR

Two studies of 138 cases and 208 controls examined the association between rs699947 polymorphism and NPDR risk. Significant association was found between rs699947 and NPDR in the dominant model (OR = 2.04, 95%CI = 1.30 − 3.21, *P* = 0.002) (Table 3).

Three studies including 366 cases and 500 controls studied the association between rs699947 polymorphism and PDR risk. The results showed that rs699947 was associated with PDR in Asian (dominant model: OR = 1.72, 95%CI = 1.05 − 2.84, *P* = 0.033; allelic model: OR = 1.48, 95%CI = 1.17 − 1.88, *P* = 0.001) (Table 4).

#### 3.2.6. *VEGF* rs13207351 and the Risk of Different Types of DR

Two studies analyzed the association between rs13207351 polymorphism and PDR risk. Due to the high heterogeneity, the summary results were that as follows: the G allele of rs13207351 was positively associated with PDR in Pakistani, while this allele had a negative association in European (Table 2).

### 3.3. Publication Bias and Sensitivity Analysis

**Figure 2:**
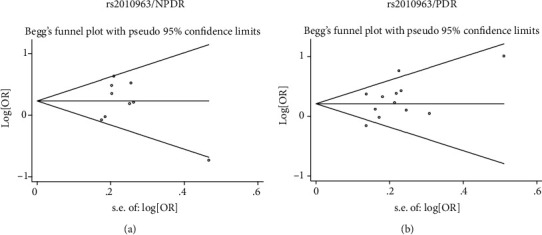
Begg's funnel plot for the association between rs2010963 and NPDR as well as PDR in the dominant model. (a) Begg's funnel plot for the association between rs2010963 and NPDR in the dominant model. (b) Begg's funnel plot for the association between rs2010963 and PDR in the dominant model.

Due to the fact that the association of rs2010963 with NPDR and PDR was conducted in 9 and 12 studies, respectively, Begg's funnel plot and the Egger's regression asymmetry test were used to assess publication bias. No publication bias was examined in the dominant model (Figure 2).

For the association between rs2010963 and NPDR as well as PDR, sensitivity analysis was used to assess the impact of each individual study on the pooled results by sequentially remove each eligible study. Deletion of any study has no significant effect on the results (Supplementary 5), indicating that the results were statistically stable and reliable. In addition, a meta-regression analysis was performed to explore the heterogeneity between studies. Variables include publication years, ethnicity, sample size, and NOS score. However, these variables have nothing to do with heterogeneity (Supplementary 3).

### 3.4. Systematic Review of Other *VEGF* SNPs and the Risk of Different Types of DR

Except the above *VEGF* gene polymorphisms, there were some other SNPs that were studied [13, 15, 28, 37–39]. Some SNPs were found to be associated with NPDR in Pakistani [13], or associated with PDR in European [37] (Supplementary 4), indicating that they may be associated with different types of DR susceptibility. However, due to the lack of research on these SNPs, their association with NPDR or PDR still needs further confirmation.

## 4. Discussion

DR is one of the most serious microvascular complications of DM, and it is the leading cause of visual impairment [40]. Evidence suggested that DR is a multifactorial disease caused by both genetic and environmental factors, in which VEGF played an important role in the development of DR [41, 42]. In this systematic review and meta-analysis, we quantitatively analyzed the association between *VEGF* gene polymorphisms and risk of different types of DR. The results showed that rs2010963 was associated with NPDR in Asian and associated with PDR in the total population, either Asian or Caucasian. Rs833061 and rs699947 polymorphisms were associated with PDR in Asian, while rs699947 was associated with NPDR risk in the total population.

*VEGF* is the main regulator of physiological and pathological vascular growth. It can induce the increase of retinal vascular permeability, the destruction of blood retinal barrier and the formation of new blood vessels in DR, which is closely associated with the occurrence and development of DR [17]. Animal study indicated that intravitreal injection of *VEGF* small interfering RNA can inhibit choroidal neovascularization and vascular permeability [43], further proving that *VEGF* is associated with DR. Studies have shown that *VEGF* gene polymorphisms, such as rs2010963, rs699947, and rs3025039, are significantly associated with serum VEGF levels [30, 44, 45], and these SNPs are associated with DR risk. However, the genetic susceptibility of NPDR and PDR is different [46]. A few studies have explored the association between *VEGF* SNPs and DR susceptibility, and some studies have conducted meta-analysis, but the results were not consistent. The most studied polymorphism of *VEGF* was rs2010963. Although most studies showed that there was no association between rs2010963 and DR, and a recent meta-analysis did not find association between them [4]. In contrast, Qiu et al. conducted a meta-analysis of studies prior to 2012 and confirmed that rs2010963 polymorphism was associated with DR [18]. Consistently, in this paper, our study indicated that rs2010963 was associated with NPDR in Asian and associated with PDR in total population, either Asian or Caucasian. These results were contrary to previous researches [46, 47], which did not find association between them. These differences may be attributed to studies of different ethnicities and different types of DR. So far, no confirmed conclusion can be drawn about the association between rs2010963 and DR, and further research is needed.

A recent meta-analysis of the association between *VEGF* gene polymorphisms and DR found that the C allele of rs833061 was positively associated with DR susceptibility [4]. Consistently, the research by Han et al. also found that the rs833061 polymorphism was associated with DR [47]. In this meta-analysis, we found that rs833061 polymorphism was associated with the development of PDR in Asian, indicating that rs833061 polymorphism was associated with PDR susceptibility. Consistent with this result, Gong et al. performed a meta-analysis of studies before 2013 and concluded that rs833061 polymorphism was associated with PDR [48]. These results indicated that rs833061 polymorphism was associated with PDR, but the role of racial factor in PDR and the association between genetic factors and different types of DR still need further study. In addition, we found that rs699947 was associated with the susceptibility of PDR in Asian. Consistent with our results, Wang et al. found that rs699947 was associated with DR in Asian populations [17]. However, Lu et al. found that rs699947 was associated with DR in European [19]. These results indicated that the genetic susceptibility of DR varies among different populations. The different results may be due to differences in the recruitment of study population and the subjects themselves, the number of studies included in meta-analysis, the type of DR, and the genetic and environmental backgrounds.

Several limitations of this study need to be considered. First, different genotyping methods used among studies may lead to inconsistent test results. Second, the inclusion of both type 1 DM and type 2 DM in some studies may have an impact on the results. Third, due to the lack of study at present, we did not analyze by country; and for SNPs with large heterogeneity between studies or studied only in one study, no conclusion can be drawn, and more exploration and research are needed to determine the association between *VEGF* gene polymorphisms and NPDR as well as PDR. Fourth, some other potential confounders, such as age, sex, duration of DM, glycemic control, genetic, and environmental interactions, may also affect the results. However, the results did not change after excluding studies containing patients with type 1 DM, and this meta-analysis increased the statistical power of the analysis, provided the latest comprehensive evidence for the association between *VEGF* gene polymorphisms and NPDR as well as PDR susceptibility. Moreover, we conducted subgroup analyses of different types of DR based on ethnicity, obtained relatively stable results through sensitivity analysis and publication bias evaluation, so the results were more credible and convincing.

## 5. Conclusions

The results indicated that rs2010963 is associated with NPDR in Asian and associated with PDR in total population, either Asian or Caucasian. Rs833061 and rs699947 polymorphisms are associated with PDR in Asian, and rs699947 is associated with the occurrence of NPDR in the total population. Considering that DR is a multifactorial disease, many environmental and genetic factors may be involved in DR, and the genetic susceptibility of different types of DR may be different in different populations. Therefore, further large-scale studies on risk factors and pathological mechanisms of different types of DR are needed in different populations.

## Figures and Tables

**Table 1:** Characteristics of studies included in the meta-analysis (NPDR versus DM).

SNPs	Author and reference	Year	Country	Ethnicity	Sample size		Mean age (years)		Male (%)		Duration of diabetes (years)		Genotype		Allele (M/m)∗		OR (95% CI)			NOS	HWE
					Case	Control	Case	Control	Case	Control	Case	Control	Case	Control	Case	Control	Dominant model	Recessive model	Allelic model		
rs2010963 and (G>C)	Khan et al. [11]	2019	Pakistan	Asian	301	573	53.43 ± 10.82	54.32 ± 10.04	43.85	47.64	12.78 ± 8.48	12.73 ± 9.23	24/34/31	41/47/23	82/96	129/93	0.49 (0.26-0.92)	0.63 (0.34-1.16)	0.62 (0.41-0.92)	6	0.170
	Kamal et al. [12]	2016	Egypt	Caucasian	15	34	54.9 ± 6.8	53.9 ± 8.6	53.33	17.65	10 (10-14)	4.5 (2.0-6.5)	7/7/1	8/20/6	21/9	36/32	0.35 (0.09-1.27)	0.33 (0.04-3.05)	0.48 (0.19-1.20)	6	0.292
	Choudhuri et al. [14]	2015	India	Asian	70	102	52 ± 8.8	53.1 ± 7.86	55.71	53.92	16.6 ± 5.8	17.9 ± 5.76	40/26/4	64/34/4	106/34	162/42	1.26 (0.68-2.35)	1.49 (0.36-6.15)	1.24 (0.74-2.07)	6	0.845
	Yuan et al. [22]	2014	China	Asian	124	144	61.10 ± 9.47	60.95 ± 9.96	48.39	48.61	8.63 ± 6.24	7.66 ± 5.90	46/52/26	49/64/31	144/104	162/126	0.88 (0.53-1.44)	0.97 (0.54-1.74)	0.93 (0.66-1.31)	7	0.244
	Yang et al. [23]	2010	China	Asian	110	109	58.98 + 1.14	52.01 + 1.06	40.00	55.96	18.0 + 0.67	15.0 + 1.20	42/53/15	49/55/5	137/83	153/65	1.32 (0.77-2.27)	3.28 (1.15-9.38)	1.43 (0.96-2.12)	6	0.032
	Chun et al. [24]	2010	Korea	Asian	108	134	59.4 ± 10.1	58.3 ± 9.5	38.89	38.81	14.4 ± 6.5	13.7 ± 6.6	37/52/19	43/69/22	126/90	155/113	0.91 (0.53-1.55)	1.09 (0.55-2.13)	0.98 (0.68-1.41)	7	0.519
	Uthra et al. [25]	2008	Indian	Asian	79	82	—	65.4 ± 8.9	—	62.20	—	19.6 ± 6.1	39/33/7	44/29/6	111/47	117/41	1.29 (0.69-2.41)	1.18 (0.38-3.69)	1.21 (0.74-1.98)	6	0.690
	Szaflik et al. [26]	2008	Poland	Caucasian	72	61	71.7 ± 11.9	67.7 ± 7.9	43.06	32.79	18.9 ± 9.8	12.4 ± 7.2	5/42/23	6/46/6	52/88	58/58	1.50 (0.43-5.19)	4.24 (1.59-11.32)	1.69 (1.03-2.79)	7	<0.001
	Awata et al. [27]	2002	Japan	Asian	80	118	61.0 ± 11.4	54.0 ± 15.1	48.75	51.69	13.0 ± 7.1	7.3 ± 6.8	14/51/15	47/59/12	79/81	153/83	3.12 (1.57-6.19)	2.04 (0.90-4.63)	1.89 (1.26-2.85)	6	0.295
rs3025039 and (C>T)	Choudhuri et al. [14]	2015	India	Asian	70	102	52 ± 8.8	53.1 ± 7.86	55.71	53.92	16.6 ± 5.8	17.9 ± 5.76	31/34/5	60/33/9	96/44	153/51	1.80 (0.97-3.32)	0.80 (0.26-2.48)	1.38 (0.85-2.22)	6	0.166
	Zhang et al. [28]	2011	China	Asian	80	92	60.88 ± 11.36	61.55 ± 10.4	48.75	47.83	9.69 ± 4.59	9.61 ± 4.48	55/20/5	46/34/12	130/30	126/58	0.46 (0.24-0.85)	0.44 (0.15-1.32)	0.50 (0.30-0.83)	7	0.167
	Kim et al. [29]	2009	South Korea	Asian	84	277	—	—	—	—	—	—	40/44/0	226/51/0	124/44	503/51	4.88 (2.88-8.24)	—	3.50 (2.24-5.48)	6	0.092
	Uthra et al. [25]	2008	Indian	Asian	86	82	—	65.4 ± 8.9	—	62.20	—	—	76/10/0	65/17/0	162/10	147/17	0.50 (0.22-1.18)	—	0.53 (0.24-1.20)	6	0.295
	Awata et al. [27]	2002	Japan	Asian	80	118	61.0 ± 11.4	54.0 ± 15.1	48.75	51.69	13.0 ± 7.1	7.3 ± 6.8	49/27/4	85/31/2	125/35	201/35	1.63 (0.89-2.98)	3.05 (0.55-17.08)	1.61 (0.96-2.70)	6	0.664
rs833061 and (T>C)	Khan et al. [11]	2019	Pakistan	Asian	301	573	53.43 ± 10.82	54.32 ± 10.04	43.85	47.64	12.78 ± 8.48	12.73 ± 9.23	34/40/18	57/71/65	108/76	185/201	2.09 (1.15-3.79)	1.40 (0.83-2.36)	1.54 (1.08-2.20)	6	<0.001
	Yuan et al. [22]	2014	China	Asian	124	144	61.10 ± 9.47	60.95 ± 9.96	48.39	48.61	8.63 ± 6.24	7.66 ± 5.90	86/34/4	78/51/15	206/42	207/81	0.52 (0.32-0.86)	0.29 (0.09-0.89)	0.52 (0.34-0.79)	7	0.137
	Szaflik et al. [26]	2008	Poland	Caucasian	72	61	71.7 ± 11.9	67.7 ± 7.9	43.06	32.79	18.9 ± 9.8	12.4 ± 7.2	3/41/27	3/34/24	47/95	40/82	1.17 (0.23-6.03)	0.95 (0.47-1.91)	0.99 (0.59-1.65)	7	0.039
	Wang et al. [30]	2008	China	Asian	65	75	60.24 ± 9.42	60.18 ± 10.26	50.77	45.33	13.56 ± 3.65	13.24 ± 3.15	10/36/19	26/33/16	56/74	85/65	2.92 (1.28-6.66)	1.52 (0.71-3.29)	1.73 (1.08-2.78)	7	0.367
rs1570360 and (G>A)	Khan et al. [11]	2019	Pakistan	Asian	301	573	53.43 ± 10.82	54.32 ± 10.04	43.85	47.64	12.78 ± 8.48	12.73 ± 9.23	37/33/23	98/58/36	107/79	254/130	0.70 (0.39-1.27)	0.63 (0.38-1.05)	0.69 (0.48-0.99)	6	<0.001
	Choudhuri et al. [14]	2015	India	Asian	70	102	52 ± 8.8	53.1 ± 7.86	55.71	53.92	16.6 ± 5.8	17.9 ± 5.76	46/21/3	72/27/3	113/27	171/33	1.25 (0.65-2.40)	1.48 (0.29-7.54)	1.24 (0.71-2.17)	6	0.809
	Chun et al. [24]	2010	Korea	Asian	108	134	59.4 ± 10.1	58.3 ± 9.5	38.89	38.81	14.4 ± 6.5	13.7 ± 6.6	74/29/5	81/46/7	177/39	208/60	0.70 (0.41-1.20)	0.88 (0.27-2.86)	0.76 (0.49-1.20)	7	0.888
	Awata et al. [27]	2002	Japan	Asian	80	118	61.0 ± 11.4	54.0 ± 15.1	48.75	51.69	13.0 ± 7.1	7.3 ± 6.8	64/15/1	88/29/1	143/17	205/31	0.73 (0.37-1.46)	1.48 (0.09-24.03)	0.79 (0.42-1.47)	6	0.403
rs699947 and (C>A)	Shahin et al. [31]	2015	Egypt	Caucasian	30	74	53.13 ± 8.44	48.57 ± 11.09	0.07	43.24	14.53 ± 3.82	12.0 ± 3.24	10/20/0	30/40/4	40/20	100/48	1.36 (0.56-3.32)	0.26 (0.01-4.92)	1.04 (0.55-1.97)	6	0.045
	Chun et al. [24]	2010	Korea	Asian	108	134	59.4 ± 10.1	58.3 ± 9.5	38.89	38.81	14.4 ± 6.5	13.7 ± 6.6	52/47/9	92/36/6	151/65	220/48	2.36 (1.40-3.99)	1.94 (0.67-5.63)	1.97 (1.29-3.02)	7	0.317

M/m∗, major/minor allele. Genotype presented as wild type/heterozygous/homozygous; −, not available; SNPs, single nucleotide polymorphisms; OR, odds ratio; 95% CI, 95% confidence interval; NOS, Newcastle–Ottawa quality assessment scale; HWE, Hardy–Weinberg equilibrium.

**Table 2:** Characteristics of studies included in the meta-analysis (PDR versus DM).

SNPs	Author and reference	Year	Country	Ethnicity	Sample size		Mean age (years)		Male (%)		Duration of diabetes(years)		Genotype		Allele (M/m)∗			OR(95% CI)		NOS	HWE
					Case	Control	Case	Control	Case	Control	Case	Control	Case	Control	Case	Control	Dominant model	Recessive model	Allelic model		
rs2010963	Khan et al. [11]	2019	Pakistan	Asian	252	573	51.9±11.36	54.32±10.04	44.44	47.64	12.01±7.69	12.73±9.23	23/28/22	38/49/24	74/72	125/97	0.64(0.33-1.26)	0.88(0.47-1.66)	0.80(0.53-1.21)	6	0.279
(G>C)	Kamal et al. [12]	2016	Egypt	Caucasian	12	34	58.1±7.5	53.9±8.6	16.67	17.65	13 (10-20)	4.5 (2.0-6.5)	2/3/7	8/20/6	7/17	36/32	1.54(0.28-8.53)	6.53(1.54-27.78)	2.73(1.00-7.43)	6	0.292
	Choudhuri et al. [14]	2015	India	Asian	105	102	53.4 ± 8.15	53.1 ± 7.86	54.28	53.92	17.2 ± 6.58	17.9 ± 5.76	47/41/17	64/34/4	135/75	162/42	2.08(1.19-3.62)	4.73(1.53-14.60)	2.14(1.38-3.33)	6	0.845
	Yuan et al. [22]	2014	China	Asian	108	144	60.66 ± 8.54	60.95 ± 9.96	37.04	48.61	11.52 ± 5.76	7.66 ± 5.90	24/56/28	49/64/31	104/112	162/126	1.81(1.02-3.19)	1.28(0.71-2.29)	1.39(0.97-1.97)	7	0.244
	Yang et al. [23]	2010	China	Asian	66	109	55.43+1.69	52.01+1.06	43.94	55.96	16.0+0.98	15.0+1.20	25/30/11	49/55/5	80/52	153/65	1.34(0.72-2.50)	4.16(1.38-12.58)	1.53(0.97-2.41)	6	0.032
	Chun et al. [24]	2010	Korea	Asian	145	134	58.6±10.6	58.3±9.5	40.00	38.81	12.9±8.2	13.7±6.6	48/73/24	43/69/22	169/121	155/113	0.96(0.58-1.58)	1.01(0.54-1.90)	0.98(0.70-1.38)	7	0.519
	Nakamura et al. [32]	2009	Japan	Asian	177	292	-	-	54.80	57.53	22.7±8.9	16.7±7.5	63/79/34	84/146/59	205/147	314/264	0.73(0.49-1.10)	0.93(0.58-1.50)	0.85(0.65-1.12)	6	0.760
	Petrovic et al. [33]	2008	Slovene	Caucasian	206	143	65.0 ± 9.9	66.9 ± 11.5	46.12	39.86	19.2 ± 8.7	16.5 ± 6.6	79/103/24	61/67/15	261/151	189/97	1.20(0.77-1.85)	1.13(0.57-2.23)	1.13(0.82-1.55)	7	0.589
	Uthra et al. [25]	2008	Indian	Asian	41	82	-	65.4 ± 8.9	-	62.20	-	19.6 ± 6.1	21/18/2	44/29/6	60/22	117/41	1.20(0.56-2.55)	0.62(0.12-3.24)	1.05(0.57-1.92)	6	0.690
	Szaflik et al. [26]	2008	Poland	Caucasian	82	61	60.7±12.9	67.7±7.9	41.46	32.79	21.5±8.8	12.4±7.2	9/56/13	6/46/6	74/82	58/58	0.89(0.30-2.64)	1.73(0.62-4.87)	1.11(0.69-1.79)	7	<0.001
	Errera et al. [34]	2007	southern Brazil of European ancestry	Caucasian	167	334	45.17±11.08	48.51 ± 10.05	40.12	60.18	15.22±8.39	12.81±7.48	57/73/37	139/155/40	187/147	433/235	1.38(0.93-2.03)	2.09(1.28-3.42)	1.45(1.11-1.89)	7	0.039
	Awata et al. [27]	2002	Japan	Asian	70	118	55.5 ±11.3	54.0 ± 15.1	50.00	51.69	12.7 ± 8.7	7.3 ± 6.8	24/30/16	47/59/12	78/62	153/83	1.27(0.69-2.35)	2.62(1.16-5.93)	1.47(0.96-2.25)	6	0.295
rs3025039	Choudhuri et al. [14]	2015	India	Asian	105	102	53.4 ± 8.15	53.1 ± 7.86	54.28	53.92	17.2 ± 6.58	17.9 ± 5.76	36/48/21	60/33/9	120/90	153/51	2.74(1.56-4.81)	2.58(1.12-5.95)	2.25(1.48-3.42)	6	0.166
(C>T)	Zhang et al. [28]	2011	China	Asian	82	92	59.96±9.89	61.55±10.4	51.22	47.83	9.37±4.51	9.61±4.48	57/20/5	46/34/12	134/30	126/58	0.44(0.24-0.82)	0.43(0.15-1.29)	0.49(0.29-0.81)	7	0.167
	Kim et al. [29]	2009	South Korea	Asian	37	277	-	-	-	-	-	-	15/19/3	226/51/0	49/25	503/51	6.50(3.15-13.40)	56.30(2.85-1113.22)	5.03(2.87-8.82)	6	0.092
	Uthra et al. [25]	2008	Indian	Asian	44	82	-	65.4 ± 8.9	-	62.20	-	-	35/9/0	65/17/0	79/9	147/17	0.98(0.40-2.43)	-	0.99(0.42-2.31)	6	0.295
	Awata et al. [27]	2002	Japan	Asian	70	118	55.5 ±11.3	54.0 ± 15.1	55.71	51.69	12.7 ± 8.7	7.3 ± 6.8	44/20/6	85/31/2	108/32	201/35	1.52(0.81-2.86)	5.44(1.07-27.73)	1.70(0.99-2.90)	6	0.664
rs833061	Khan et al. [11]	2019	Pakistan	Asian	252	573	51.9±11.36	54.32±10.04	44.44	47.64	12.01±7.69	12.73±9.23	32/24/23	57/71/65	88/70	185/201	1.24(0.70-2.19)	1.62(0.94-2.80)	1.37(0.94-1.98)	6	<0.001
(T>C)	Yuan et al. [22]	2014	China	Asian	108	144	60.66 ± 8.54	60.95 ± 9.96	37.04	48.61	11.52 ± 5.76	7.66 ± 5.90	63/36/9	78/51/15	162/54	207/81	0.84(0.51-1.40)	0.78(0.33-1.86)	0.85(0.57-1.27)	7	0.137
	Paine et al. [35]	2012	India	Asian	253	240	52±15.0	54±12.0	52.56	53.33	15±8	17±5	152/81/20	167/67/6	385/121	401/79	1.52(1.05-2.21)	3.35(1.32-8.49)	1.60(1.16-2.19)	7	0.814
	Szaflik et al. [26]	2008	Poland	Caucasian	82	61	60.7±12.9	67.7±7.9	41.46	32.79	21.5±8.8	12.4±7.2	2/44/36	3/34/24	48/116	40/82	2.07(0.34-12.78)	1.21(0.62-2.37)	1.18(0.71-1.96)	7	0.039
	Wang et al. [30]	2008	China	Asian	64	75	65.52±8.78	60.18±10.26	48.44	45.33	14.94±2.85	13.24±3.15	9/37/18	26/33/16	55/73	85/65	3.24(1.39-7.59)	1.44(0.66-3.14)	1.74(1.08-2.79)	7	0.367
rs1570360	Khan et al. [11]	2019	Pakistan	Asian	252	573	51.9±11.36	54.32±10.04	44.44	47.64	12.01±7.69	12.73±9.23	26/20/27	98/58/36	72/74	254/130	0.39(0.22-0.72)	0.53(0.30-0.93)	0.50(0.34-0.73)	6	<0.001
(G>A)	Choudhuri et al. [14]	2015	India	Asian	105	102	53.4 ± 8.15	53.1 ± 7.86	54.28	53.92	17.2 ± 6.58	17.9 ± 5.76	58/40/7	72/27/3	156/54	171/33	1.95(1.10-3.45)	2.36(0.59-9.38)	1.79(1.11-2.91)	6	0.809
	Chun et al. [24]	2010	Korea	Asian	145	134	58.6±10.6	58.3±9.5	40.00	38.81	12.9±8.2	13.7±6.6	92/49/4	81/46/7	233/57	208/60	0.88(0.54-1.43)	0.52(0.15-1.80)	0.85(0.56-1.28)	7	0.888
	Churchill et al. [36]	2008	European Caucasians	Caucasian	45	61	59.6(24-86)	55.3(24-97)	60.00	52.46	23.1(7-44)	24.6(14-50)	4/15/26	24/28/9	23/67	76/46	6.65(2.11-20.96)	7.91(3.14-19.89)	4.81(2.65-8.76)	7	0.858
	Awata et al. [27]	2002	Japan	Asian	70	118	55.5 ±11.3	54.0 ± 15.1	50.00	51.69	12.7 ± 8.7	7.3 ± 6.8	50/17/3	88/29/1	117/23	205/31	1.17(0.60-2.28)	5.24(0.53-51.37)	1.30(0.72-2.33)	6	0.403
rs699947	Shahin et al. [31]	2015	Egypt	Caucasian	44	74	51.18 ± 15.31	48.57 ± 11.09	50.00	43.24	16.36 ± 5.55	12.0 ± 3.24	12/32/0	30/40/4	56/32	100/48	1.82(0.81-4.09)	0.18(0.01-3.35)	1.19(0.68-2.07)	6	0.045
(C>A)	Chun et al.[24]	2010	Korea	Asian	145	134	58.6±10.6	58.3±9.5	40.00	38.81	12.9±8.2	13.7±6.6	71/68/6	92/36/6	210/80	220/48	2.28(1.40-3.72)	0.92(0.29-2.93)	1.75(1.17-2.62)	7	0.317
	Nakamura et al. [32]	2009	Japan	Asian	177	292	-	-	54.80	57.53	22.7±8.9	16.7±7.5	85/70/22	163/107/22	240/114	433/151	1.37(0.94-1.99)	1.74(0.93-3.25)	1.36(1.02-1.82)	6	0.449
rs13207351	Khan et al. [11]	2019	Pakistan	Asian	252	573	51.9±11.36	54.32±10.04	44.44	47.64	12.01±7.69	12.73±9.23	20/24/30	73/60/60	64/84	206/180	1.64(0.91-2.96)	1.51(0.87-2.63)	1.50(1.03-2.20)	6	<0.001
(A>G)	Churchill et al. [36]	2008	European Caucasians	Caucasians	45	61	59.6(24-86)	55.3(24-97)	60.00	52.46	23.1(7-44)	26.4(14-50)	29/7/9	21/29/11	65/25	71/51	0.29(0.13-0.65)	1.14(0.43-3.03)	0.54(0.30-0.96)	7	0.858

Mm∗: major/minor allele. Genotype presented as wild type/heterozygous/homozygous; −, not available; SNPs, single nucleotide polymorphisms; OR, odds ratio; 95% CI, 95% confidence interval; NOS, Newcastle–Ottawa quality assessment scale; HWE, Hardy–Weinberg equilibrium.

**Table 3:** Meta-analysis of the associations between *VEGF* SNPs and NPDR.

SNPs	Number of study	Sample size		Dominant model				Recessive model				Allelic model			
		Case	Control	OR (95% CI)	*I* ^2^ (%)	*P*_*H*_	*P*_*Z*_	OR (95% CI)	*I* ^2^ (%)	*P*_*H*_	*P*_*Z*_	OR (95% CI)	*I* ^2^ (%)	*P*_*H*_	*P*_*Z*_
rs2010963															
Total	9	959	1357	1.25 (1.01-1.54)	45.3	0.067	0.039	1.58 (1.20-2.09)	37.5	0.119	0.001	1.26 (1.02-1.56)∗	50.6	0.040	0.031
Asian	7	872	1262	1.29 (1.04-1.60)	44.5	0.094	0.023	1.48 (1.11-1.99)	11.5	0.342	0.009	1.26 (1.08-1.47)	43.5	0.101	0.003
Caucasian	2	87	95	0.73 (0.18-3.04)∗	60.5	0.112	0.534	—	76.7	0.038	—	—	82.1	0.018	—
rs1570360															
Total (Asian)	4	559	927	0.80 (0.59-1.09)	0.0	0.515	0.158	0.72 (0.47-1.12)	0.0	0.714	0.142	0.80 (0.63-1.01)	0.0	0.395	0.062
rs699947															
Total	2	138	208	2.04 (1.30-3.21)	7.5	0.298	0.002	1.36 (0.54-3.44)	39.4	0.199	0.519	1.50 (0.81-2.79)∗	62.4	0.103	0.199

SNPs, single nucleotide polymorphisms; *I*^2^, Higgins *I^2^* statistic; *P*_*H*_, value for heterogeneity test; *P*_*Z*_, value for the *Z* test; OR, odds ratio; CI, confidence interval; −, not available. ∗ORs were calculated in random-effect model.

**Table 4:** Meta-analysis of the associations between *VEGF* SNPs and PDR.

SNPs	Number of study	Sample size		Dominant model				Recessive model				Allelic model			
		Case	Control	OR (95% CI)	*I* ^2^ (%)	*P*_*H*_	*P*_*Z*_	OR (95% CI)	*I* ^2^ (%)	*P*_*H*_	*P*_*Z*_	OR (95% CI)	*I* ^2^ (%)	*P*_*H*_	*P*_*Z*_
rs2010963															
Total	12	1431	2126	1.20 (1.03-1.41)	17.6	0.271	0.022	1.66 (1.21-2.28)∗	50.7	0.022	0.002	1.23 (1.11-1.37)	49.0	0.028	<0.001
Asian	8	964	1554	1.17 (0.97-1.43)	43.7	0.087	0.108	1.57 (1.04-2.35)∗	54.7	0.030	0.030	1.26 (1.01-1.57)∗	58.9	0.017	0.039
Caucasian	4	467	572	1.27 (0.96-1.67)	0.0	0.872	0.090	1.83 (1.28-2.63)	42.4	0.157	0.001	1.31 (1.09-1.57)	23.9	0.268	0.004
rs833061															
Total	5	759	1093	1.41 (0.94-2.11)∗	50.7	0.088	0.094	1.49 (1.09-2.04)	27.5	0.238	0.013	1.33 (1.11-1.58)	46.3	0.114	0.002
Asian	4	677	1032	1.40 (0.90-2.17)∗	62.0	0.048	0.138	1.58 (1.11-2.26)	41.1	0.165	0.012	1.34 (0.99-1.81)∗	58.4	0.066	0.056
Caucasian	1	82	61	2.07 (0.34-12.78)	—	—	0.434	1.21 (0.62-2.37)	—	—	0.585	1.18 (0.71-1.96)	—	—	0.524
rs699947															
Total	3	366	500	1.67 (1.27-2.21)	26.1	0.259	<0.001	1.32 (0.78-2.23)	31.8	0.231	0.304	1.44 (1.16-1.78)	0.0	0.481	0.001
Asian	2	322	426	1.72 (1.05-2.84)∗	62.4	0.103	0.033	1.50 (0.87-2.60)	0.0	0.342	0.145	1.48 (1.17-1.88)	0.0	0.328	0.001
Caucasian	1	44	74	1.82 (0.81-4.09)	—	—	0.148	0.18 (0.01-3.35)	—	—	0.248	1.19 (0.68-2.07)	—	—	0.537

SNPs, single nucleotide polymorphisms; *I*^2^, Higgins *I*^2^ statistic; *P*_*H*_, value for heterogeneity test; *P*_*Z*_ value for the *Z* test; OR, odds ratio; CI, confidence interval; −, not available. ∗ORs were calculated in random-effect model.

## Data Availability

The data used to support the findings of this study are included within the article and supplementary information files.

## Supplementary Materials

Supplementary 1 PRISMA checklist.

Supplementary 2 Literature Search Strategy.

Supplementary 3 Meta-regression analysis for the association between rs2010963 and NPDR as well as PDR.

Supplementary 4 Other SNPs of *VEGF* gene identified in different populations.

Supplementary 5 Sensitivity analysis of the association of rs2010963 with NPDR and PDR in the dominant model. (a) Sensitivity analysis of the association of rs2010963 with NPDR in the dominant model. (b) Sensitivity analysis of the association of rs2010963 with PDR in the dominant model.
