# Supplementary material for: Associations between Vascular Endothelial Growth Factor Gene Polymorphisms and Different Types of Diabetic Retinopathy Susceptibility: A Systematic Review and Meta-Analysis
Source: J Diabetes Res. 2021 Jan 4;2021:7059139. doi: 10.1155/2021/7059139 (PMC7805525; doi:10.1155/2021/7059139)
Supplement: Supplementary 2 — Literature Search Strategy. [file 7059139.f2.docx]

| **Table S2.** Literature Search Strategy | | |
| --- | --- | --- |
| Pubmed | | |
| 1 | Search ((diabetic retinopathy*[Title/Abstract]) OR diabetes retinopathy*[Title/Abstract]) OR DR*[Title/Abstract] | 108749 |
| 2 | Search ((vascular endothelial growth factor*[Title/Abstract]) OR vascular endothelial cell growth factor*[Title/Abstract]) OR VEGF*[Title/Abstract] | 83783 |
| 3 | Search (((((gene*[Title/Abstract]) OR polymorphism*[Title/Abstract]) OR mutation*[Title/Abstract]) OR single nucleotide polymorphism*[Title/Abstract]) OR SNP*[Title/Abstract]) OR variant*[Title/Abstract] | 2255119 |
| 4 | 1 AND 2 AND 3 | 343 |
| 5 | Filters: Humans | 223 |
| Embase | | |
| 1 | ('diabetic retinopathy*':ab,ti OR 'diabetes retinopathy*':ab,ti) AND [embase]/lim | 25458 |
| 2 | ('vascular endothelial growth factor*':ab,ti OR 'vascular endothelial cell growth factor*':ab,ti OR 'vegf*':ab,ti) AND [embase]/lim | 112279 |
| 3 | ('gene*':ab,ti OR 'polymorphism*':ab,ti OR 'mutation*':ab,ti OR 'single nucleotide polymorphism*':ab,ti OR 'snp*':ab,ti OR 'variant*':ab,ti) AND [embase]/lim | 5239904 |
| 4 | #1 AND #2 AND #3 | 705 |
| 5 | #1 AND #2 AND #3 AND [humans]/lim | 439 |
| Web of Science | | |
| 1 | TS=("diabetic retinopathy*") OR TS=("diabetes retinopathy*") OR TS=("DR")  Databases= WOS, BCI, KJD, RSCI, SCIELO Timespan=All years Search language=Auto | 128825 |
| 2 | TS=("vascular endothelial growth factor*") OR TS=("vascular endothelial cell growth factor*") OR TS=(VEGF*)  Databases= WOS, BCI, KJD, RSCI, SCIELO Timespan=All years Search language=Auto | 136285 |
| 3 | TS=("gene*") OR TS=("polymorphism*") OR TS=(mutation*) OR TS=("single nucleotide polymorphism*) OR TS=("SNP*") OR TS=(variant*")  Databases= WOS, BCI, KJD, RSCI, SCIELO Timespan=All years Search language=Auto | 19928673 |
| 4 | #1 AND #2 AND #3  Databases= WOS, BCI, KJD, RSCI, SCIELO Timespan=All years Search language=Auto | 5224 |
| 5 | #4 NOT TS=(animal)  Databases= WOS, BCI, KJD, RSCI, SCIELO Timespan=All years Search language=Auto | 347 |
| CNKI | | |
| 1 | 糖尿病视网膜病变 或者 DR | 72650 |
| 2 | 血管内皮生长因子 或者 血管内皮细胞生长因子 或者 VEGF | 86907 |
| 3 | 基因 或者 多态性 或者 突变 或者 单核苷酸多态性 或者 SNP 或者 变体 | 2094786 |
| 4 | #1 AND #2 AND #3 | 271 |
| WANFANG | | |
| 1 | 糖尿病视网膜病变+DR | 453525 |
| 2 | 血管内皮生长因子+血管内皮细胞生长因子+VEGF | 130223 |
| 3 | 基因+多态性+突变+单核苷酸多态性+SNP+变体 | 2534378 |
| 4 | #1 AND #2 AND #3 | 620 |
| 5 | #4人 | 388 |
