# Supplementary material for: Associations between Vascular Endothelial Growth Factor Gene Polymorphisms and Different Types of Diabetic Retinopathy Susceptibility: A Systematic Review and Meta-Analysis
Source: J Diabetes Res. 2021 Jan 4;2021:7059139. doi: 10.1155/2021/7059139 (PMC7805525; doi:10.1155/2021/7059139)
Supplement: Supplementary 3 — Meta-regression analysis for the association between rs2010963 and NPDR as well as PDR. [file 7059139.f3.docx]

| **Table S3.** Meta-regression analysis for the association between rs2010963 and NPDR as well as PDR. | | | | | | | |
| --- | --- | --- | --- | --- | --- | --- | --- |
| Variable | NPDR | | |  | PDR | | |
|  | Coefficient | *t* | *P* |  | Coefficient | *t* | *P* |
| Publication Year | -0.0466 | -1.52 | 0.172 |  | 0.0209 | 0.85 | 0.414 |
| Ethnicity | -0.5680 | -1.01 | 0.344 |  | 0.0317 | 0.14 | 0.893 |
| Sample size | 0.0004 | 0.60 | 0.570 |  | -0.0005 | -0.91 | 0.382 |
| NOS score | -0.4504 | -1.62 | 0.149 |  | 0.0508 | 0.24 | 0.813 |
