# Supplementary material for: Associations between Vascular Endothelial Growth Factor Gene Polymorphisms and Different Types of Diabetic Retinopathy Susceptibility: A Systematic Review and Meta-Analysis
Source: J Diabetes Res. 2021 Jan 4;2021:7059139. doi: 10.1155/2021/7059139 (PMC7805525; doi:10.1155/2021/7059139)
Supplement: Supplementary 4 — Other SNPs of VEGF gene identified in different populations. [file 7059139.f4.docx]

| Table S4. Other SNPs of *VEGF* gene identified in different populations | | | | | | | | | | | | | | | | | | | | | | | | |  |
| --- | --- | --- | --- | --- | --- | --- | --- | --- | --- | --- | --- | --- | --- | --- | --- | --- | --- | --- | --- | --- | --- | --- | --- | --- | --- |
| SNPs | Author and reference | Year | Country | comparison(1=NPDR&DM, 2=PDR&DM) | Sample size | |  | Mean age (years) | | Male (%) | | Duration of diabetes(years) | | Genotype | | Allele (M/m)* | | OR (95% CI) | | | | | NOS | HWE |  |
|  |  |  |  |  | Case | Control |  | Case | Control | Case | Control | Case | Control | Case | Control | Case | Control | Dominant model |  | Recessive model |  | Allelic model |  |  |  |
| rs13207351 (A>G) | Khan et al.[11] | 2019 | Pakistan | 1 | 301 | 573 |  | 53.43±10.82 | 54.32±10.04 | 43.85 | 47.64 | 12.78±8.48 | 12.73±9.23 | 19/44/30 | 73/60/60 | 82/104 | 206/180 | 2.37(1.32-4.24) |  | 1.06(0.62-1.80) |  | 1.45(1.02-2.06) | 6 | <0.001 |  |
| rs10738760 (A>G) | Sajovic et al.[37] | 2019 | Slovenian | 2 | 143 | 362 |  | 63.4±9.5 | 64.7±10.2 | 48.95 | 44.48 | 18.8±6.2 | 11.8±2.6 | 32/81/30 | 87/186/89 | 145/141 | 360/364 | 1.10(0.69-1.74) |  | 0.81(0.51-1.30) |  | 0.96(0.73-1.27) | 6 | 0.599 |  |
| rs2071559 (A>G) | Choudhuri et al.[14] | 2015 | India | 1 | 70 | 102 |  | 52±8.8 | 53.1±7.86 | 55.71 | 53.92 | 16.6±5.8 | 17.9±5.76 | 34/27/9 | 44/40/18 | 95/45 | 128/76 | 0.80(0.44-1.48) |  | 0.69(0.29-1.64) |  | 0.80(0.51-1.26) | 6 | 0.104 |  |
| *VEGF* (D/I) | Li et al.[38] | 2013 | China | 1 | 30 | 31 |  | 63±9.6 | 62±9.3 | 46.67 | 45.16 | 13±5.1 | 12±4.2 | 16/11/3 | 12/11/8 | 43/17 | 35/27 | 0.48(0.17-1.34) |  | 0.32(0.08-1.35) |  | 0.47(0.22-1.01) | 7 | 0.121 |  |
| *VEGF* (D/I) | Li et al.[38] | 2013 | China | 2 | 30 | 31 |  | 65±9.2 | 62±9.3 | 43.33 | 45.16 | 15±5.6 | 12±4.2 | 17/10/3 | 12/11/8 | 44/16 | 35/27 | 0.55(0.20-1.53) |  | 0.32(0.08-1.35) |  | 0.51(0.24-1.09) | 7 | 0.121 |  |
| rs2146323 (C>A) | Churchill et al.[36] | 2008 | European | 2 | 45 | 61 |  | 59.6(24-86) | 55.3(24-97) | 60.00 | 52.46 | 23.1(7-44) | 26.4(14-50) | 19/26/0 | 26/22/13 | 64/26 | 74/48 | 1.02(0.47-2.22) |  | 0.04(0.01-0.68) |  | 0.63(0.35-1.12) | 7 | 0.056 |  |
| rs735286 (C>T) | Churchill et al. [36] | 2008 | European | 2 | 45 | 61 |  | 59.6(24-86) | 55.3(24-97) | 60.00 | 52.46 | 23.1(7-44) | 26.4(14-50) | 10/35/0 | 32/27/2 | 55/35 | 91/31 | 3.86(1.63-9.16) |  | 0.26(0.01-5.58) |  | 1.87(1.04-3.36) | 7 | 0.190 |  |
| VEGF-160 (C>T) | Churchill et al. [36] | 2008 | European | 2 | 45 | 61 |  | 59.6(24-86) | 55.3(24-97) | 60.00 | 52.46 | 23.1(7-44) | 26.4(14-50) | 43/2/0 | 41/20/0 | 88/2 | 102/20 | 0.10(0.02-0.43) |  | - |  | 0.12(0.03-0.51) | 7 | 0.126 |  |
| T( 1498)C-G( 1190)A | Awata et al.[27] | 2002 | Japan | 1 | 80 | 118 |  | 61.0±11.4 | 54.0±15.1 | 48.75 | 51.69 | 13.0±7.1 | 7.3±6.8 | 42/33/5 | 52/57/9 | 117/43 | 161/75 | 0.71(0.40-1.26) |  | 0.81(0.26-2.51) |  | 0.79(0.51-1.23) | 6 | 0.215 |  |
| T( 1498)C-G( 1190)A | Awata et al.[27] | 2002 | Japan | 2 | 70 | 118 |  | 55.5±11.3 | 54.0±15.1 | 50.00 | 51.69 | 12.7±8.7 | 7.3±6.8 | 37/25/8 | 52/57/9 | 99/41 | 161/75 | 0.70(0.39-1.27) |  | 1.56(0.57-4.26) |  | 0.89(0.56-1.40) | 6 | 0.215 |  |
| G1612A | Awata et al.[27] | 2002 | Japan | 1 | 80 | 118 |  | 61.0±11.4 | 54.0±15.1 | 48.75 | 51.69 | 13.0±7.1 | 7.3±6.8 | 59/20/1 | 92/22/4 | 138/22 | 206/30 | 1.26(0.65-2.44) |  | 0.36(0.04-3.29) |  | 1.10(0.61-1.98) | 6 | 0.083 |  |
| G1612A | Awata et al.[27] | 2002 | Japan | 2 | 70 | 118 |  | 55.5±11.3 | 54.0±15.1 | 50.00 | 51.69 | 12.7±8.7 | 7.3±6.8 | 57/10/3 | 92/22/4 | 124/16 | 206/30 | 0.81(0.38-1.70) |  | 1.28(0.28-5.88) |  | 0.89(0.46-1.69) | 6 | 0.083 |  |
| C(-7)T (C>T) | Awata et al.[27] | 2002 | Japan | 1 | 80 | 118 |  | 61.0 ± 11.4 | 54.0± 15.1 | 48.75 | 51.69 | 13.0± 7.1 | 7.3± 6.8 | 55/22/3 | 78/35/5 | 132/28 | 191/45 | 0.89(0.48-1.63) |  | 0.88(0.20-3.79) |  | 0.90(0.54-1.52) | 6 | 0.672 |  |
| C(-7)T (C>T) | Awata et al.[27] | 2002 | Japan | 2 | 70 | 118 |  | 55.5 ± 11.3 | 54.0± 15.1 | 50.00 | 51.69 | 12.7± 8.7 | 7.3± 6.8 | 50/20/0 | 78/35/5 | 120/20 | 191/45 | 0.78(0.41-1.49) |  | 0.15(0.01-2.69) |  | 0.71(0.40-1.26) | 6 | 0.672 |  |
| M/m*: major/minor allele | | | | | | | | | | | | | | | | | | | | | | | | | |
| Genotype presented as wild type/heterozygous/homozygous; −, not available; SNPs: single nucleotide polymorphisms; OR: odds ratio; 95% CI: 95% confidence interval; NOS: Newcastle–Ottawa quality assessment scale; HWE: Hardy–Weinberg equilibrium. | | | | | | | | | | | | | | | | | | | | | | | | | |
