# Supplementary figures and images for: Associations between Vascular Endothelial Growth Factor Gene Polymorphisms and Different Types of Diabetic Retinopathy Susceptibility: A Systematic Review and Meta-Analysis
Source: J Diabetes Res. 2021 Jan 4;2021:7059139. doi: 10.1155/2021/7059139 (PMC7805525; doi:10.1155/2021/7059139)

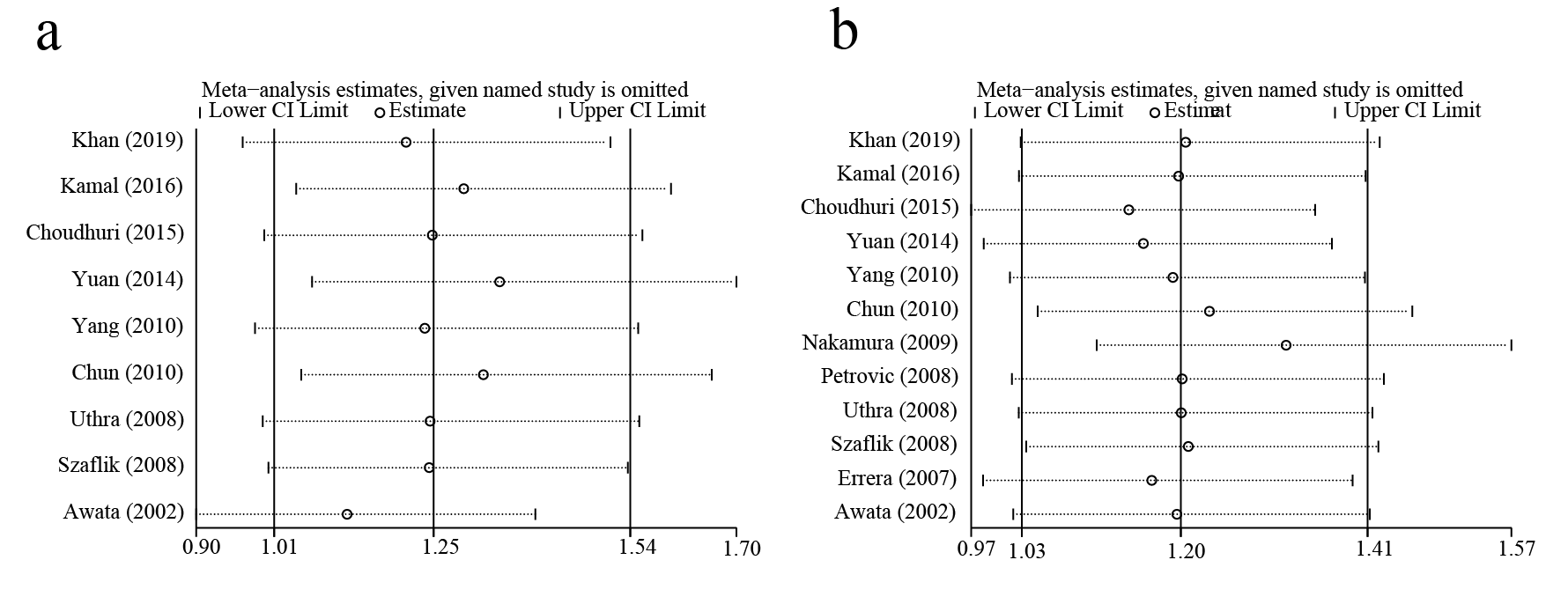

Supplement: Supplementary 5 — Sensitivity analysis of the association of rs2010963 with NPDR and PDR in the dominant model. (a) Sensitivity analysis of the association of rs2010963 with NPDR in the dominant model. (b) Sensitivity analysis of the association of rs2010963 with PDR in the dominant model. [file 7059139.f5.tif]
